# Studies on the Spatiotemporal Variability of River Water Quality and Its Relationships with Soil and Precipitation: A Case Study of the Mun River Basin in Thailand

**DOI:** 10.3390/ijerph15112466

**Published:** 2018-11-05

**Authors:** Zhonghe Zhao, Gaohuan Liu, Qingsheng Liu, Chong Huang, He Li

**Affiliations:** 1 State Key Laboratory of Resources and Environmental Information System, Institute of Geographic Sciences and Natural Resources Research, Chinese Academy of Sciences, Beijing 100101, China; zhaozh.16b@igsnrr.ac.cn (Z.Z.); huangch@lreis.ac.cn (C.H.); lih@lreis.ac.cn (H.L.); 2 University of Chinese Academy of Sciences, Beijing 100049, China

**Keywords:** soil nutrients, water quality, precipitation

## Abstract

Human activities can affect soil nutrients, thereby influencing river water quality. The spatial pattern of precipitation also impacts distributions of water quality. In this paper, we employed a method that combines point survey, soil, and water quality data to analyze the spatial relationships between precipitation, soil nutrient and water quality in the basin on the basis of field surveys and laboratory analysis. The ordinary kriging method was applied to interpolate the precipitation and soil data, and the spatial pattern was analyzed. The water samples on the main stream and soil samples in the field were collected during both the dry and rainy seasons to analyze the water quality and soil nutrients. The results indicate: (1) The water quality in the dry season is better than that in the rainy season, the water quality in the upper reaches is better than that in the lower reaches, and agricultural activity is the direct source of water pollution. (2) The precipitation in the rainy and dry seasons is differente and the dilution effect of precipitation on pollutant concentrations and transport of water flow affect the spatial distribution of water quality. (3) There is a significant difference in the spatial pattern of soil nutrients between the dry and rainy seasons, and the soil nutrient content and the surface runoff directly affect the water quality. Soil nutrients are affected by human activities, and they potentially act as nonpoint source (NPS) pollution in this river basin. To improve the water quality, suitable agriculture measures need to be implemented.

## 1. Introduction

Land use affects the emission and transport of pollutants and has an important influence on river water quality [1]. However, the multiscale and different distribution patterns of land use have led to uncertainty with regard to river water quality based on the land use types [2]. As the issue of water pollution is becoming increasingly serious, the evaluation of water quality has become more important. The evaluation of water quality involves the theories, methods, and application of water quality science, which is an important branch of environmental assessment and serves as the basis to understand and protect aquatic environments [3]. Water quality evaluations have a long history; early evaluations were mainly conducted to qualitatively describe the sensory properties of water, such as color, taste, smell, and turbidity. Along with the continuous development of science and technology, relatively deep understanding of the physical, chemical, and biological properties of water bodies were improved, and meanwhile, multiple water quality evaluation methods were developed. With the introduction of mathematical models, new water quality evaluation methods have continuously emerged, including the index evaluation method [3,4,5,6,7] and water quality evaluation methods based on the fuzzy mathematics theory, gray theory, multivariate statistics, and artificial neural networks [8,9,10,11]. Of these, multivariate statistical methods, such as factor analysis (FA), clustering analysis (CA), and discriminant analysis (DA), have been widely applied to sociological, education, and medical research, and gradually been applied to research on water quality [12]. In particular, the application of principal component analysis (PCA) is relatively broad [13,14].

Soil pollution can be referred to as nonpoint source (NPS) pollution, and it can also be called diffused pollution. NPS pollution refers to water pollution caused by dissolved or solid pollutants flowing into rivers, lakes, reservoirs, and oceans during precipitation events and from runoff erosion. The main sources of NPS pollution include urban runoff, hydrological variations, agricultural production runoff, irrigation return flows, solid waste, atmospheric deposition, and riverbed erosion. At present, two types of NPS pollution that are of particular concern for water pollution are agricultural/rural NPS pollution and urban NPS pollution [15].

NPS pollution has received widespread attention over the past 40 years. In 1987, the United States (US) revised Article 319 of the Clean Water Act to clarify NPS pollution regulations. However, agricultural/rural NPS pollution is still an important problem affecting US water quality. According to the US national water quality survey in 2000, the water qualities of 39% of river segments, 45% of lakes, and 51% of estuaries were damaged to varying degrees, which was mainly attributed to the complicated problem of NPS pollution.

This study aims to study the spatial and seasonal corelations between water quality and precipitation, water quality and soil nutrients contents. The following contents were addressed: the characteristics of water quality in the dry and rainy seasons in the Mun River Basin, the spatial distribution patterns of precipitation in the dry and rainy seasons and their relationship with the water quality, and the spatial distribution pattern of soil nutrients and its relationship with the water quality.

Changes in soil nutrient contents are the consequence of the interaction between natural factors and human factors, and changes in land use type inevitably cause changes in soil nutrient content. By understanding the differences in the soil nutrient contents of different crop cultivations, we can provide the basis for the rational utilization of land resources and protection of the ecological environment [13,14]. Further more, the latent NPS pollution potential is high in the region and could effect the water quality. Understanding this relations will promote the future restoration and improvement of the ecological environment in the Mun River Basin.

## 2. Materials and Methods

### 2.1. General Information of the Study Area

**Figure 1:**
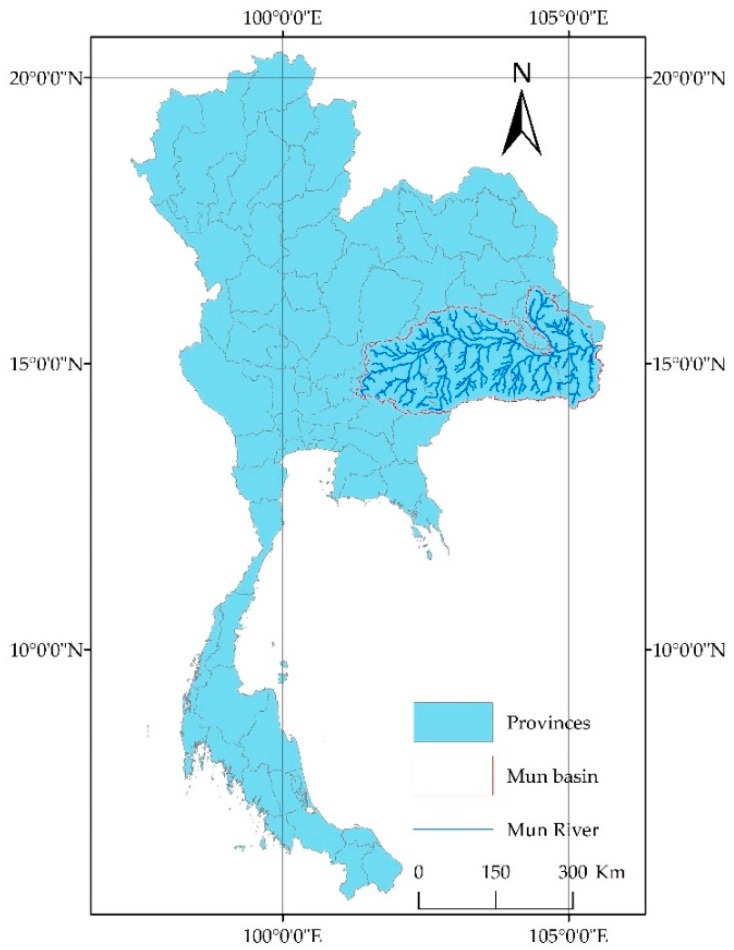
Location of the Mun River Basin.

The Mun River is the largest river on the Korat Plateau in northeastern Thailand and is one of the important tributaries of the Mekong River, showed as Figure 1. The Mun River is approximately 673 km long and has a river basin area of approximately 82,000 km^2^. Within the basin, the terrain is high in the west and low in the east; plateaus and mountains are in the southwestern area, and plains are in the central and eastern areas. In the southwestern highlands, mountains and rivers are alternately distributed, with most of them running in a south-north direction in a vertically distributed manner, creating rugged terrain. In the central and eastern regions, many plains and rivers run in the south-north direction, which provides sufficient water resources for water cycling in these plain regions.

The Mun River Basin has a humid subtropical climate, and the climate is most significantly affected by the tropical monsoons in Asia. The climate and hydrology within the basin show significant seasonal differences changes brought upon by the seasonal monsoon render distinct wet and dry seasons in the basin. The annual temperature is not lower than 18 °C, and the average annual rainfall is 1300–1500 mm.

In summer, the southwest monsoon blowing from the Indian Ocean generates high temperatures and abundant rainfall. Given the frequent rainfall during this time, summer is generally referred to as the “rainy season”. The rainy season lasts from mid-May to the beginning of October, with heavy rainfall generally concentrated in August or September. In winter, due to the strong Mongolian cold and high pressure, the northeast monsoon brings low temperatures and dry weather, and this period is generally referred to as the “dry season”. The dry season lasts from November to April of the following year, with an average temperature of 16 °C (high temperature can be up to 40–42 °C). The transitional season is between the dry season and the rainy season, and frequent nondirectional winds occur at this time. As the dry season and rainy season result from the monsoon effect, an extremely uneven distribution of rainfall exists during the year.

### 2.2. Data and Methods

#### 2.2.1. Water Quality Data

**Figure 2:**
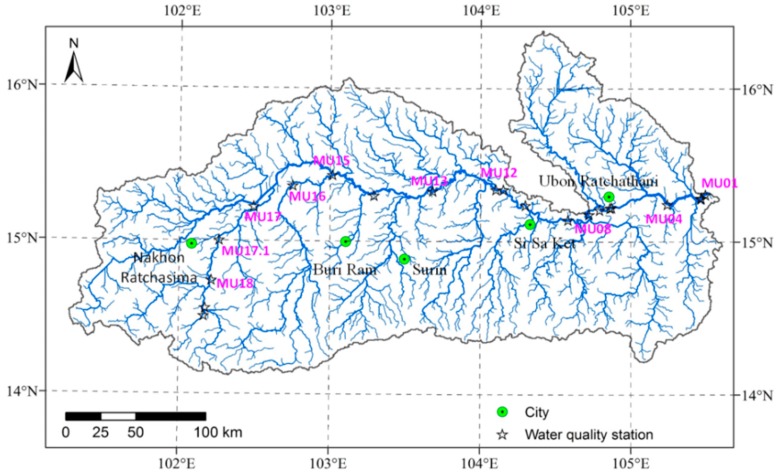
Distribution of the water quality monitoring stations in the Mun River.

Two sets of water quality data were derived from 19 water quality monitoring stations in the main stream of the Mun River in 2017, one in the rainy season (August) and the other in the dry season (February). The location of the 19 water quality monitoring stations is shown in Figure 2. We adopted 10 water quality indexes in this sduty, namely, ammoniacal nitrogen (NH_3_-N), nitrate nitrogen (NO_3_-N), nitrite nitrogen (NO_2_-N), total phosphorus (TP), dissolved oxygen (DO), biochemical oxygen demand (BOD), EC, pH, turbidity (nephelometric turbidity units, NTU), and suspended solid (SS). In particular, DO, BOD, EC, NTU, and pH were measured in situ by a U-5000 (HORIBA, Kyoto, Japan) multiparameter water quality meter; polyethylene plastic bottles (1 L) were used to collect the water samples, which were used to test TP, NO_2_-N, NH_3_-N, and NO_3_-N. The water samples were titrated by sulfuric acid to pH < 2 and placed inside the storage cabin at a temperature < 4 °C. The water samples were then sent to the laboratory to test the water quality. In particular, phosphorus content (mg/L) was measured using the vanadomolybdophosphoric acid method, NO_3_-N content (mg/L) was measured using the brucine method, NH_3_-N (mg/L) content was measured using the titration method, NO_2_-N content was measured using diazamine coincidence spectrophotometry, and the determination of SS was completed using the gravimetric method [16].

#### 2.2.2. Precipitation Data

**Figure 3:**
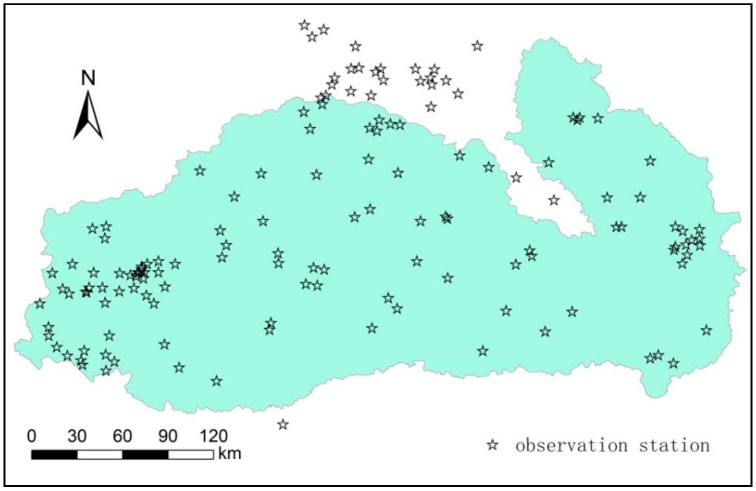
Location map of precipitation observation stations in the basin and surrounding areas.

Based on a long-term time series of daily precipitation observational data, statistical analysis of annual average precipitation in the basin from 1960–2015 was performed. The precipitation trends during the dry season (November–April of next year) and rainy season (May–October) were also calculated. There are a total of 144 precipitation observation stations throughout the basin (Figure 3). To ensure the accuracy of the interpolation results, data from the observation stations in the marginal region of the basin were retained. By combining geostatistics and kriging interpolation, the spatial patterns of annual average precipitation and precipitation in the dry and rainy seasons were calculated for the entire basin.

#### 2.2.3. Soil Data

**Figure 4:**
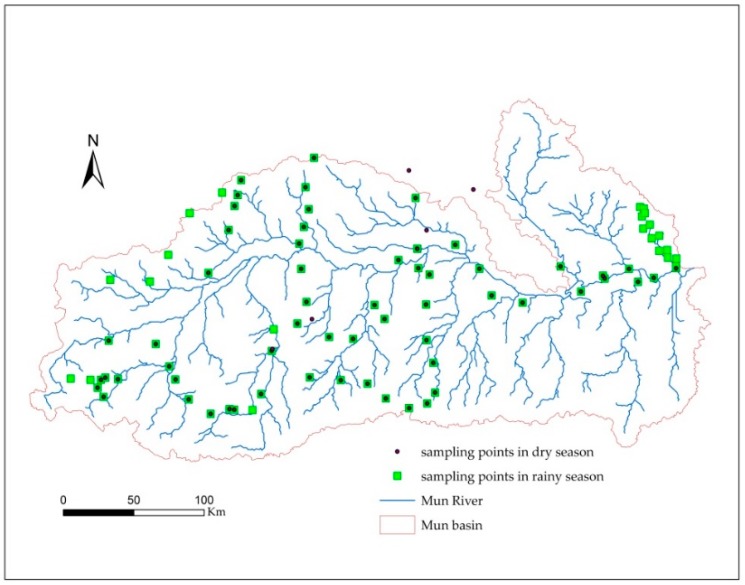
Location map of soil sampling points.

In this study, we mainly analyze the spatial patterns and variability of soil nutrients, pH, and EC in the dry and rainy seasons. Therefore, a consistent sampling method was adopted, different soil and land use types were considered. The sampling times were February of 2017 (dry season) and August of 2017 (rainy season), and soil samples were collected from representative sampling points. After the soil was evenly mixed, it was divided by coning and quartering; samples of approximately 1.5 kg of soil were retained and dried in the laboratory. The plant roots, leaves, and stones were removed from the residual soil. To ensure the consistency of the sampling locations, the slope of every sampling site is <5° and GPS was used to record the longitude and latitude coordinates of each sampling point. The vegetation coverage status, land use and soil moisture were recorded and photographed for each sampling point. The soil sampling depth is 0–20 cm and total 66 samples in the dry season and 86 samples in the rainy season were celected. The specific location of the soil sampling points is shown in Figure 4.

### 2.3. Data Processing

The water quality data were statistically analyzed according to PCA using Microsoft Excel (Microsoft, Redmond, WA, USA) and SPSS software (IBM, Armonk, NY, USA); the best-fit interpolation model was selected for soil nutrient and precipitation data by GS+ 7.0 (Gamma Design Software, LLC, Plainwell, MI, USA), and then the spatial interpolation was completed in ArcGIS (Esri, Redlands, CA, USA).

The PCA method belongs to the FA category [17], and it is a statistical analysis method to identify the principal correlations among multiple variables. PCA can analyze the main influencing factors, reveal relationships, and simplify complex questions. The goal for calculating the principal component involves projecting high-dimensional data to relatively low-dimensional space [18]. The PCA used in water quality evaluation has two main aspects: (1) establish a comprehensive evaluation index, evaluate the relative pollution levels between different sampling sites and analyze the pollution level at various sampling sites; and (2) evaluate the role of each index within a comprehensive index, remove the secondary indexes, and determine the main compositions of pollution.

In this study, by selecting the optimum interpolation model for SOM, nitrogen, phosphorus, pH, and EC, we employed ordinary kriging interpolation and analyze the spatiotemporal variability of soil within the research area. In particular, the optimal model and the spatial interpolation parameters were selected using GS+ statistical software, and kriging spatial interpolation was performed using ArcGIS software.

The semivariance function of samples was calculated using the formula:

(1) $$\;\gamma \left(h\right)=\;\frac{1}{2N\left(h\right)}\overset{N\left(h\right)}{\underset{i=1}{\sum }}[Z\left(x_{i}+h\right)-Z\left(x_{i}\right){]}^{2}\;$$

where γ(h) is the semivariance of the samples, h is the distance between two sampling points (also referred to as the lag distance), N(h) is the number of paired data at a distance of the interval h, and $Z\left(x_{i}+h\right)\;\mathrm{and}\;Z\left(x_{i}\right)$ are the measured values at the sampling point of $x_{i}+h$ and of $x_{i}$, respectively. The semivariance scatter plots calculated from the actual sampling points need to be fitted using the semivariance model to obtain the spatially related semivariance curves. In this study, the gaussian model, spherical model, exponential model and linear model were used to fit the optimal model.

## 3. Results and Analysis

### 3.1. PCA of Water Quality

#### 3.1.1. Characteristics of Water Quality in the Dry Season

The PCA of water quality was completed in SPSS. The results of the correlation coefficient matrix are as given in Table 1:

The table shows the correlation coefficient matrix for various water quality indexes. Larger absolute values of the correlation coefficients between two indexes indicate stronger correlations between the two indexes. The correlation coefficient between NO_3_-N and NH_3_-N is the highest (0.902), followed by that of NO_2_-N and NO_3_-N (0.825) and that of NH_3_-N and NTU (0.662).

The principle used to extract the number of principal components corresponds to the first principal components with eigenvalues greater than 1. An eigenvalue is an index that expresses the influence of the principal component. An eigenvalue less than 1 indicates that the interpretation strength of this principal component is not sufficient. The cumulative contribution rate of the first three principal components in Table 2 reaches 78.30%; that is, these three principal components can explain most of the data variability. Therefore, we select these three principal components for further analysis.

The load matrix of the principal components indicates the relationships between different indexes and the principal components. If the absolute value of the correlation coefficient between the index and a certain principal component is high, the principal component is closely connected to the index. The Table 3 shows that the loads for the indexes of pH, EC, NO_3_-N, NO_2_-N, NH_3_-N, and NTU in the first principal component are relatively high, which indicates that the first principal component reflects the information of these indexes, i.e., mainly human activities and NPS pollution. The loads of DO, BOD, and SS in the second principal component are also relatively high, which indicates that the second principal component mainly reflects the information of these three indexes, i.e., mainly sediment pollution. The loads of TP and SS in the third principal component are relatively high, which indicates that the third principal component mainly reflects the information of these two indexes, i.e., mainly water eutrophication pollution.

The principal component load matrix for each water quality index was used to calculate the principal component score and comprehensive score of 19 water quality monitoring points (Table 4), which quantified and described the degree of water quality at each station. The higher the score, the more serious the pollution degree was. Therefore, the pollution degree of each monitoring station could be analyzed.

**Figure 5:**
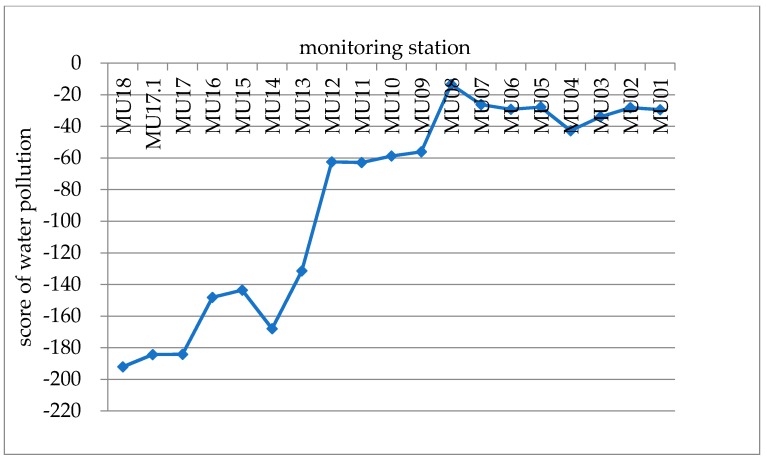
Comprehensive score of water pollution during the dry season.

Figure 5 indicates that the water quality gradually declines from the upper reaches to the lower reaches. In particular, the water quality is highest in the upper reach MU18–MU13, and the water quality gradually decreases from MU12.

#### 3.1.2. Characteristics of Water Quality in the Rainy Season

The correlation coefficient matrix for various indexes of water quality is shown in Table 5. In the rainy season, the correlations between pH and TP and NO_2_-N are relatively similar, i.e., 0.587 and 0.507, respectively; the correlations between EC and DO; salt content and NO_2_-N and NH_3_-N; and TP and NH_3_-N, NO_3_-N, and NO_2_-N and the correlations among NH_3_-N, NO_3_-N, and NO_2_-N are also relatively similar.

The cumulative contribution rate of the first four principal components in the PCA (Table 6) of water quality index reached 78.03%; means these four principal components can explain most of the data variability. Therefore, these four principal components were selected for water quality extraction and analysis.

Table 7 shows that the loads for the indexes of pH, EC, NO_2_-N, NH_3_-N, SS, and TP on the first principal component are relatively high, which indicates that the first principal component reflects these indexes. The loads of DO, NO_3_-N, and BOD are also relatively high in the second principal component, which indicates that the second principal component reflects these indexes. The loads of NO_3_-N, NTU, and SS are relatively high in the third principal component, which indicates that the third principal component reflects these indexes. The loads of pH, BOD, and SS are relatively high in the fourth principal component, which indicates that the fourth principal component mainly reflects these indexes.

**Figure 6:**
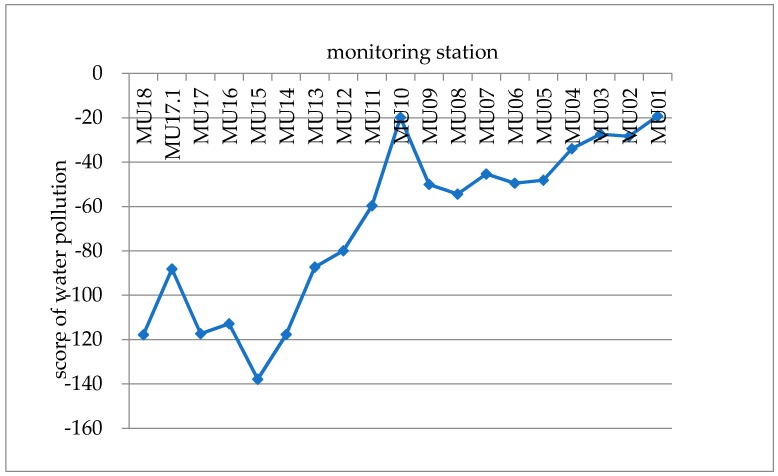
Comprehensive score of water pollution in the rainy season.

Lastly, the principal component score and comprehensive pollution score of the 19 water quality monitoring stations were calculated and the water pollution levels at different stations were quantitatively described. Higher scores indicate a greater degree of pollution. Therefore, we can analyze the pollution level at each monitoring station, and the results are shown in Table 8:

Figure 6 shows that the water quality in the rainy season was generally lower than that in the dry season, and this difference is mainly related to the enhancement of agricultural cultivation and pollutant emissions during the rainy season. In the rainy season, the water quality was relatively high in the upper reaches, and from the upper reaches to the lower reaches, the water quality gradually declined. In particular, the water quality of MU18–MU13 was relatively high, and the water quality of MU12–MU01 was relatively poor.

### 3.2. Spatial Distribution of Precipitation

The parameters provided by the geostatistical analysis further reveal the characteristics of the spatial distribution of precipitation throughout the Mun River Basin. The nugget value indicates the random variation likely caused by human activities and sampling errors. The sill value indicates the total variation in precipitation exhibited under the current scale. Table 9 lists the geostatistical analysis results of model parameters of precipitation in the Mun River Basin.

There are 144 precipitation stations, and the distribution of these stations is uniform; thus, the statistical results are relatively robust. We selected the optimal model by the GS+ calculation. In particular, the relevant indexes of the exponential model of annual average precipitation are as follows: the nugget value is 8800, the sill value is 41,850, and the spatial heterogeneity ratio is 0.790, which indicates that the spatial correlation of the annual average precipitation is relatively weak. The optimal model of annual average precipitation is the exponential model.

The optimal model of the dry season precipitation is the spherical model. The nugget value is 119, the sill value is 439.9, and the spatial heterogeneity ratio is 0.729, which suggests moderate spatial correlation; the maximum correlation distance is 0.632 m.

The optimal model of the rainy season precipitation is the exponential model. The nugget value is 7700, the sill value is 52,280, and the spatial heterogeneity ratio is 3.426, which indicates that the spatial correlation is relatively weak; the maximum correlation distance is 0.853, and the spatial correlation distance is larger than that of the dry season.

**Figure 7:**
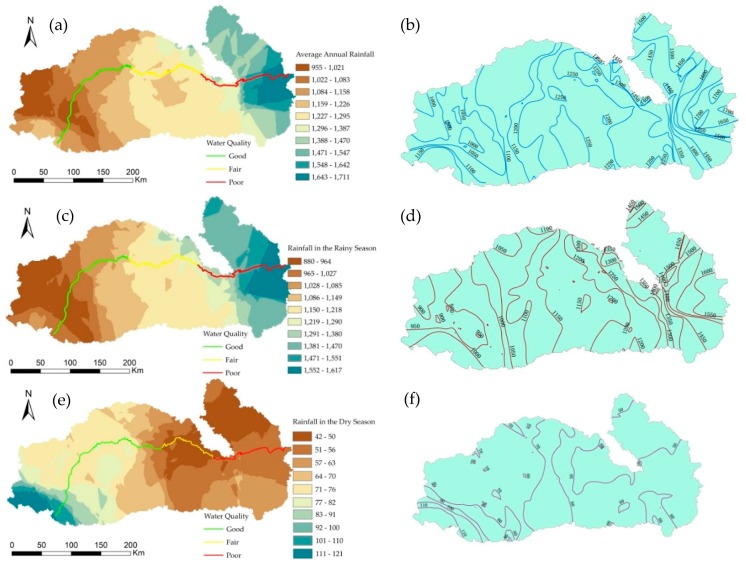
Spatial patterns of annual precipitation in the Mun River Basin. (**a**) is the map of average annual rainfall; (**b**) is the map of average annual rainfall contour; (**c**) is the map of rainfall in the rainy season; (**d**) is the map of rainfall contour in the rainy season; (**e**) is the map of rainfall in the dry season; (**f**) is the map of rainfall contour in the dry season.

From the upper reaches to the lower reaches of the Mun River, the annual average precipitation gradually increased (Figure 7a,b). The maximum precipitation reaches 1711 mm, and the minimum precipitation is 955 mm. Based on the precipitation contours, we can observe a “vortex” shape in the upper, middle, and lower reaches.

The distribution pattern and variation trend for precipitation in the rainy season in the Mun River Basin are similar to those of the annual precipitation (Figure 7c,d), which is because most of the annual precipitation occurs in the rainy season. The spatial distribution trend during the rainy season gradually increases from the upper reach to the lower reach; the maximum precipitation is as high as 1617 mm, and the minimum precipitation is 880 mm.

The spatial distribution of precipitation in the dry season is opposite to that in the rainy season as the precipitation gradually decreases from the upper reaches to the lower reaches (Figure 7e,f). In the upper reaches, most precipitation occurs in the southwest, which is directly related to the topography. The elevation in the southwest region is relatively high, and it is mainly covered by forest, which is conducive to precipitation. The precipitation is relatively low in the middle and lower reaches, and it exhibits an uneven step-like decrease, which is likely related to the vegetation and topographic conditions.

A comprehensive analysis of the spatial interpolation results of precipitation and the PCA of water quality indicates that the water quality in the upper reaches is relatively high, the water quality in the middle reach is average, and the water quality in the lower reach is the lowest. This pattern is related to precipitation, i.e., high amounts of precipitation can dilute pollutants, thereby reducing the pollutant concentration; however, due to the transport of water flow, pollutants are transported from the upper reach to the lower reach, and the pollutant concentration in the lower reach can in turn increase as pollutants accumulate.

### 3.3. Spatial Distribution Pattern of Soil Nutrients and Its Relation with Water Quality

#### 3.3.1. Spatial Distribution of Soil Nutrients and Its Relation with Water Quality in the Dry Season

**Figure 8:**
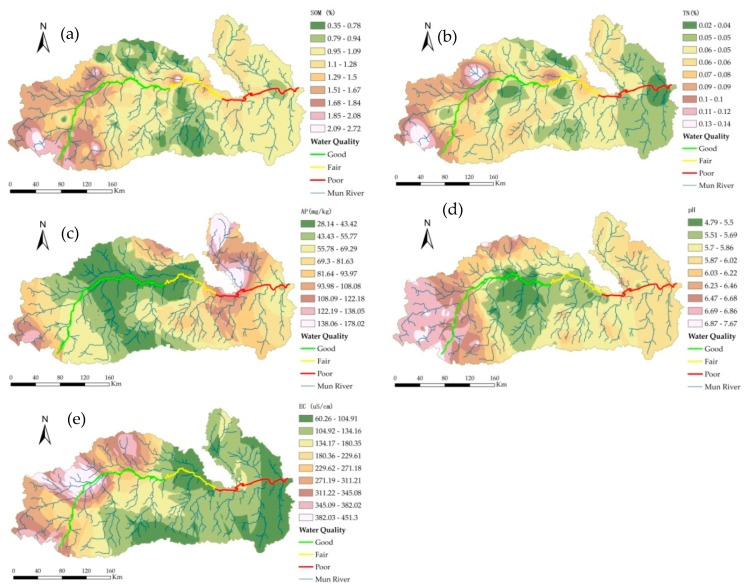
Spatial patterns of (**a**) SOM, (**b**) TN, (**c**) AP, (**d**) pH and (**e**) EC in the dry season.

Based on the semivariance function model and related parameters, the ordinary kriging method was used to perform optimal spatial interpolation for SOM, TN content, AP, pH, and EC in the dry season. Moreover, the spatial distributions of SOM, TN content, AP, pH, and EC were mapped (Figure 8), providing an intuitive description of the spatial distribution of the soil characteristics in the study area.

The interpolation results for SOM were affected by various systems or random factors and showed complex spatial variability; however, the interpolation results could still explain the patterns of the SOM distribution. The SOM in the dry season showed a concave distribution as a whole, with patchy distributions in some areas. The central plains had a flat terrain and were mostly farmland, and the SOM content was highest in these areas; the areas of the upper reaches had relatively high terrain and were mostly dry land and forestland, and the SOM content was high; the lower reaches had the lowest SOM content.

The TN content was significantly correlated with the SOM, AP, and soil sand content at the 0.05 level. The spatial distribution of the soil TN content was similar to that of SOM, showing that the spatial correlation of TN and SOM was strong. The soil TN content was medium to high in some regions of the upper and lower reaches, whereas it was low in the middle reaches of the Mun Basin.

The soil AP content had a spatial distribution that was low in the middle reaches and was medium and high in the upper and lower reaches. In the middle reaches, the irrigation conditions were good with a sufficient water supply and two to three crop growth seasons per year; the soil was subjected to great interference by human activities, and crop uptake and flooding had great impacts on the AP content. The spatial distribution of AP was mainly related to fertilization, cycling, and mobility of phosphorus.

The distribution of the pH values showed a pattern of relatively low pH values in the middle reaches, which may be related to the leaching of calcium and magnesium ions due to the high amounts of rainfall in the central region. The areas with low soil pH were also conducive to the accumulation of SOM. The upper reaches had high pH values; due to the sandy soil, the amount of evaporation exceeded the amount of precipitation, which resulted in serious soil salinization. The pH values in the lower reaches were relatively high.

The distribution of EC throughout the study area showed a trend of high in the west and low in the east, which was closely related to land use/planting patterns, geographical factors, and climatic factors.

The relationships between soil and water quality based on Pearson correlation analysis are shown in Table 10. The comprehensive score of water quality in the Mun River is significantly correlated with SOM (0.05 level), with a Pearson coefficient of 0.483. Water quality is also significantly correlated with soil total nitrogen (TN) (0.01 level), with a Pearson coefficient of 0.783, and soil EC (0.01 level), with a Pearson coefficient of 0.638. The EC of water is significantly correlated with SOM, soil TN, and soil EC. The TP content of water and soil EC are significantly correlated (0.05 level), with a Pearson coefficient of 0.468. NO_3_-N of water is significantly correlated with soil available phosphorus (AP), soil TN, and soil EC. NO_2_-N and NH_3_-N of water are significantly correlated with soil AP and soil EC, respectively.

#### 3.3.2. Spatial Distribution of Soil Nutrients and Its Relation with Water Quality in the Rainy Season

**Figure 9:**
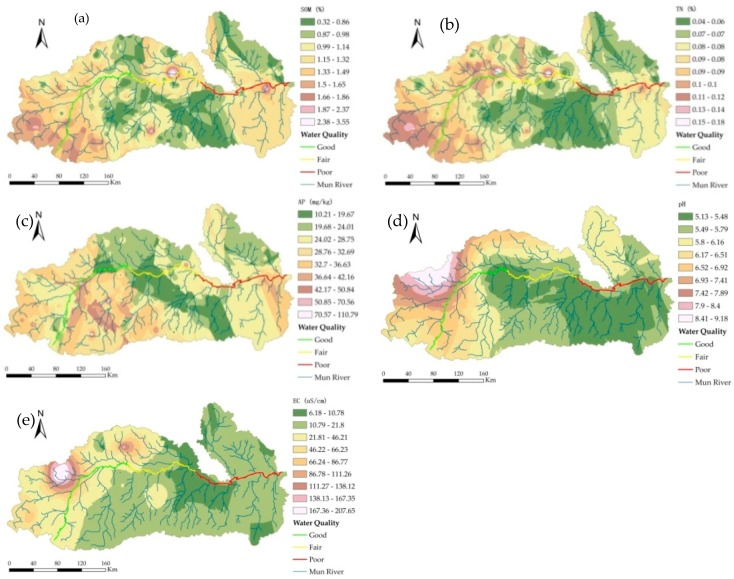
Spatial patterns of (**a**) SOM, (**b**) TN, (**c**) AP, (**d**) pH, and (**e**) EC in the rainy season.

Ordinary kriging was used to perform optimal spatial interpolation on the SOM, TN content, AP, pH, and EC in the rainy season based on the obtained semivariance function model and related parameters, and the spatial distributions of SOM, TN content, AP, pH, and EC in the study area were graphed (Figure 9) to intuitively describe the spatial distribution of the soil properties in the Mun River Basin. 

The spatial pattern of SOM in the rainy season was similar to that in the dry season, and SOM was relatively stable within a year. The SOM content in the upper and lower reaches was medium and high, whereas that in the middle reaches of the region was low.

TN and SOM content were significantly correlated at the 0.05 level, and the spatial distribution of soil TN was similar to that of SOM, indicating that the spatial correlation between TN and SOM was strong. The TN content was moderate to high in the upper and lower reaches and was relatively low in the middle reaches.

The spatial pattern of AP showed adequate similarities with the patterns in the dry season, with both seasons showing a pattern of relatively low in the central region yet high in the upper and lower reaches. The spatial distribution of AP in the rainy season had a close relation with fertilization, uptake, and utilization by crops as well as the flow of surface water and river water.

The spatial distribution of pH was generally similar to that in the dry season, albeit with differences in some localities. The pH value in the middle reaches was low, and the pH in the upper reaches was high, followed by the lower reaches.

The spatial pattern of EC was similar to that observed in the dry season, showing a trend of high in the upper reaches and low in the lower reaches. EC is influenced by factors such as salt content, moisture, temperature, SOM content, and soil texture.

In the Mun River Basin, there are correlations between water quality and soil in the rainy season as showed in Table 11. In particular, the comprehensive scores of water quality and soil EC are significantly correlated (0.05 level), with a Pearson coefficient of 0.507. Water quality is also significantly correlated with the soil pH (0.05 level), with a Pearson coefficient of 0.487. The pH values of water and soil are significantly correlated (0.01 level), with a correlation coefficient of −0.472. The TP of water is significantly correlated with the soil TN, soil EC, and soil pH. The NH_3_-N of water and soil AP are significantly correlated. The NH_2_-N of water is significantly correlated with SOM, soil TN, soil EC, and soil pH value. The NH_3_-N of water is significantly correlated with soil TN, soil EC, and soil pH.

The above analysis indicates that from the upper reaches to the lower reaches of the Mun River Basin, the pollution index of water quality exhibits an increasing trend, which is consistent with the gradient of EC in the basin. That is, the EC in the upper reaches is low, and the EC in the lower reach is high. The contents of other nutrients exhibit step-like distributions in the direction of water flow. Precipitation is continuously injected from the upper reaches, supplying large amounts of nutrients, which causes the gradual increase in the water pollution in the lower reach. In addition, the degree of variation in the soil nutrient contents and water quality during the rainy season is more dramatic than that in the dry season, which is mainly related to human activities. Crop growth, fertilization, and the use of pesticides in the rainy season are more intense than those in the dry season, and all of these activities can affect soil nutrients and water quality.

## 4. Discussion

In the analysis of soil nutrients, and based on other scholars’ research [19,20,21,22], this study has the following shortcomings: first of all, the spatial distributions of soil nutrients described here are based on data from only two sets of field samplings, and no continuous, long-term monitoring data are available. Second, the soil nutrient absorbing capacities of different crop types were not considered. Some studies indicated that paddy fields have different nutrient absorption and utilization capabilities. Different crop types have various effects on the acidity or basicity of the soil. Some studies indicated that paddy fields are suitable for weakly alkaline environments, whereas dry land is suitable for weakly acidic environments. Lastly, there is also a difference in the soil nutrient content under different farming modes; under the various planting modes of dry land, paddy fields, fallow fields, and forested land, the differences in the residual nutrient elements of soil are relatively large.

The influence of human activities on soil and water quality is not negligible. In this study, we qualitatively study only the relationships between water quality and soil nutrients and precipitation, without considering the interference of human activities. Through the field investigation and household survey, we found that the type and amont of fertilizers used in the Mun River Basin are quite different; most farmers apply fertilizer according to previous experience without scientific guidance or considering the relationship between soil residual nutrients and postfertilization conditions. This practice not only could affect the imbalance of soil nutrients but also could affect the crop yield. Irregular water utilization by the residents in the Mun River Basin is observed: according to a survey, the rivers are used by residents for irrigation during the high flow periods, and the water consumption is unknown. The aforementioned shortage of data and information could affect the accuracy of this study.

For the water quality study, only the water around each monitoring station was analyzed, but without considering the confluence process. Because the influence of confluence on the water quality and soil nutrients was not considered, the qualitative analysis of the water quality according to only the limited monitoring data could affect the accuracy of the research result.

In this study, we adopted a geostatistical analysis method. We first calculated the sill value, partial sill value, and range, and then adopt kriging interpolation to interpolate the precipitation and soil nutrient contents throughout the basin according to the sampling points. Although the simulation results of this method are obviously improved in comparison with the results of other methods, the simulation for the entire basin still has some uncertainties because of the limited number of sampling points for the soil nutrients. We should increase the number of sampling points while ensuring the representativeness of the sampling points and further correctly conduct a qualitative analysis.

## 5. Conclusions

By combining a field investigation with water quality data and soil nutrients data, we quantitatively and intuitively described the relationships between precipitation, soil nutrient content and water quality in the Mun River Basin through the spatial kriging interpolation and PCA method. The main conclusions and findings are as follows:

(1) The SOM, TN, AP, pH, and EC in the Mun River Basin exhibited obvious spatial variations. According to the analysis of the differences between the dry season and rainy season, the output potential of NPS pollution in the soil is relatively high, and the land use types and fertilizer dosage have relatively significant effects on the soil nutrient content. Generally, for soil nutrient content in the dry season, except for AP and EC, whose changes are obvious, the changes of SOM, TN, and pH are expressed more subtly, and the changes are slightly higher in the upper reaches than those in the lower reaches.

There are various factors that affect the soil nutrient content. For example, the SOM is mainly subject to the influence of animal and plant residues and microbial activities. The microorganisms in the soil have an extremely important relationship with the soil nutrient content and thereby determine the nutrient content and material circulation of the soil. The nitrogen content in soil is mainly affected by human activities, including cultivation, fertilization, and irrigation, which affect the cycling and decomposition of nitrogen. The phosphorus content in soil is mainly affected by the parent material, land use types, and erosion. This study found that the nitrogen, phosphorus, and organic matter contents in the soil are not only affected by the various factors of fertilization, irrigation, and field tillage but also affected by seasonal factors. The TN in the rainy season is higher than that in the dry season, which is likely related to fertilization. The SOM and AP contents in the dry season are higher than those in the rainy season, which is mainly related to the soil texture and human interference. As the area of cultivated land rapidly increases, the latent NPS pollution potential remains high in the region and could become the limiting factor for the future restoration and improvement of the ecological environment in the Mun River Basin.

(2) Precipitation in the Mun River Basin displays a marked seasonal difference. The annual average precipitation increases from the upper reaches to the lower reaches, and the trend of precipitation in the rainy season is the same as that of annual precipitation. Precipitation in the dry season gradually declines from the upper reaches to the lower reaches, which is opposite to that in the rainy season and annually. The differences in precipitation are related to topographic factors; the elevation in the upper reach is relatively high, and the terrain in the middle and lower reaches is flat. In the dry season when the precipitation is relatively low, the upper reach region is more prone to precipitation than the other regions. However, the difference in the precipitation is also related to meteorological factors.

(3) The water quality is affected by precipitation and soil nutrient content. Precipitation can increase the confluence into rivers and dilute the pollutants in rivers; however, it can increase the surface runoff, which can transport soil nutrients to rivers. During the dry season, the precipitation in the upper reaches is higher than that in the lower reaches, whereas during the dry season, the soil nutrient content in the upper reaches is higher than that in the lower reaches. The maximum precipitation in the upper reach during the dry season is only 121 mm, which is not sufficient to form adequate surface runoff, and hence, fewer soil nutrients are transported into rivers. If the precipitation is high, the pollutant concentration in rivers could decline; therefore, the water quality in the upper reaches is relatively high in comparison with the lower reaches. In the lower reach, the precipitation is even lower, and the pollutant concentration could increase, thereby causing the lower water quality in the lower reaches.

In the rainy season, the amount of precipitation is sufficient to allow surface runoff. A large amount of soil nutrients flows into rivers. Both precipitation and the soil nutrient content are relatively high in the upper reach. The surface runoff collects and transports nitrogen and phosphorus into rivers, and therefore, the water quality in the upper reach region is relatively poor. Due to the confluence of water flow and the high precipitation in the lower reaches (higher than the precipitation in the upper reaches), more water and sediments are concentrated in the lower reaches. Especially the rainy season is the cultivating season, more fertilizer are used and transported to river, the water quality in the lower reaches is lower than that in the upper reach. This result is likely because the addition of soil nutrient is relatively greater than the addition of water.

In this study, we found that the water qualiy has an obvious seasonal difference, and such difference was caused by different factors. The spatial interpolation of soil and precipitation can be used for analysis of water quality distributions. The main NPS pollution is the agricultural activities. The results can induce a further study in future for quantitative analysis of relations betwwen water quality and agriculture management. For example, water pollution contribution percentage of fertilizer, water quality change caused by crop type, rational agriculture measures for reducing water pollution.

## Figures and Tables

**Table 1:** Correlation coefficient matrix for each water quality index.

Item	pH	EC	DO	BOD	TP	NO_3_-N	NO_2_-N	NH_3_-N	NTU	SS
**pH**	1	−0.008	−0.589	0.172	−0.469	0.523	0.456	0.419	0.266	0.136
**EC**		1.000	−0.331	0.334	0.229	−0.640	−0.573	−0.652	−0.330	0.378
**DO**			1.000	−0.272	0.389	−0.354	−0.214	−0.236	−0.035	−0.253
**BOD**				1.000	−0.141	−0.133	−0.001	−0.192	0.017	0.491
**TP**					1.000	−0.589	−0.446	−0.486	−0.202	0.144
**NO_3_-N**						1.000	0.825	0.902	0.451	−0.059
**NO_2_-N**							1.000	0.801	0.772	0.055
**NH_3_-N**								1.000	0.662	−0.006
**NTU**									1.000	0.216
**SS**										1.000

**Table 2:** The extracted principal components of water quality indexes.

Component	Initial Eigenvalue			Extract Sum of Squares and Load		
	Total	Variance %	Accumulation %	Total	Variance %	Accumulation %
**1**	4.307	43.067	43.067	4.307	43.067	43.067
**2**	2.244	22.443	65.510	2.244	22.443	65.510
**3**	1.278	12.785	78.295	1.278	12.785	78.295
**4**	0.725	7.246	85.541			
**5**	0.474	4.738	90.279			
**6**	0.409	4.088	94.367			
**7**	0.316	3.156	97.523			
**8**	0.149	1.487	99.009			
**9**	0.084	0.837	99.846			
**10**	0.015	0.154	100.000			

**Table 3:** Load matrix of the principal components of each water quality index.

	Component	1	2	3
Item				
**pH**		0.597	0.503	−0.287
**EC**		−0.611	0.665	−0.067
**DO**		−0.347	−0.722	0.379
**BOD**		−0.066	0.720	0.235
**TP**		−0.635	−0.204	0.458
**NO_3_-N**		0.941	−0.060	−0.129
**NO_2_-N**		0.915	−0.028	0.243
**NH_3_-N**		0.932	−0.141	0.089
**NTU**		0.678	−0.004	0.581
**SS**		−0.015	0.666	0.602

**Table 4:** Main component score and comprehensive pollution score.

	Component	F1	F2	F3	Pollution Score
Monitoring Station					
**MU18**		−834.45	951.48	−56.72	−192.09
**MU17.1**		−760.00	846.55	−74.08	−184.35
**MU17**		−754.74	836.04	−72.12	−184.19
**MU16**		−620.10	694.93	−53.81	−148.14
**MU15**		−590.45	654.90	−54.74	−143.58
**MU14**		−694.83	772.36	−61.28	−167.97
**MU13**		−597.95	694.46	−24.35	−131.38
**MU12**		−266.07	299.56	−19.13	−62.53
**MU11**		−266.92	298.84	−17.42	−62.93
**MU10**		−250.39	281.05	−16.08	−58.78
**MU09**		−239.91	269.93	−15.38	−56.13
**MU08**		−144.56	213.49	25.48	−13.52
**MU07**		−160.45	210.04	6.89	−26.23
**MU06**		−168.50	215.85	5.24	−29.24
**MU05**		−166.32	215.99	6.35	−27.82
**MU04**		−196.83	231.56	−10.54	−42.79
**MU03**		−155.29	182.50	−9.62	−34.02
**MU02**		−128.90	151.38	−7.56	−28.20
**MU01**		−143.25	172.49	−4.36	−29.47

**Table 5:** Correlation coefficient matrix for each water quality index.

Item	pH	EC	DO	BOD	TP	NO_3_-N	NO_2_-N	NH_3_-N	NTU	SS
**pH**	1.000	0.377	−0.090	0.048	0.587	0.158	−0.507	−0.406	0.100	−0.261
**EC**		1.000	−0.742	0.084	0.322	0.090	−0.578	−0.481	−0.362	−0.288
**DO**			1.000	0.112	−0.128	−0.402	0.144	0.077	0.205	0.239
**BOD**				1.000	0.223	−0.130	−0.546	−0.295	−0.005	−0.383
**TP**					1.000	0.008	−0.526	−0.465	−0.315	−0.233
**NO_3_-N**						1.000	0.188	0.290	0.079	−0.195
**NO_2_-N**							1.000	0.792	0.109	0.464
**NH_3_-N**								1.000	0.128	0.177
**NTU**									1.000	0.004
**SS**										1.000

**Table 6:** Variance decomposition of the extracted principal components of water quality indexes.

Component	Initial Eigenvalues			Extract Sum of Squares and Load		
	Total	Variance %	Accumulation %	Total	Variance %	Accumulation %
**1**	3.566	35.655	35.655	3.566	35.655	35.655
**2**	1.823	18.233	53.889	1.823	18.233	53.889
**3**	1.310	13.103	66.992	1.310	13.103	66.992
**4**	1.104	11.035	78.027	1.104	11.035	78.027
**5**	0.857	8.573	86.600			
**6**	0.504	5.039	91.639			
**7**	0.354	3.537	95.176			
**8**	0.307	3.068	98.244			
**9**	0.112	1.119	99.363			
**10**	0.064	0.637	100.000			

**Table 7:** Load matrix of the principal components of each water quality index.

	Component	1	2	3	4
Item					
**pH**		−0.634	0.011	0.318	0.617
**EC**		−0.755	0.457	−0.259	−0.090
**DO**		0.399	−0.805	0.094	0.182
**BOD**		−0.440	−0.462	0.265	−0.556
**TP**		−0.703	−0.075	−0.059	0.366
**NO_3_-N**		0.012	0.705	0.508	0.066
**NO_2_-N**		0.899	0.290	−0.049	0.087
**NH_3_-N**		0.761	0.336	0.200	−0.120
**NTU**		0.278	−0.211	0.710	0.195
**SS**		0.531	−0.093	−0.506	0.417

**Table 8:** Main component score and comprehensive pollution score.

	Component	F1	F2	F3	F4	Pollution Score
Monitoring Station						
**MU18**		−300.44	176.34	−96.14	−26.97	−117.76
**MU17.1**		−224.86	133.00	−79.01	−13.32	−88.14
**MU17**		−299.82	178.19	−100.15	−24.14	−117.34
**MU16**		−300.20	177.47	−78.55	−15.58	−112.81
**MU15**		−350.19	209.18	−121.79	−29.57	−137.82
**MU14**		−299.01	175.54	−100.38	−24.69	−117.69
**MU13**		−222.75	129.92	−73.28	−16.28	−87.32
**MU12**		−204.16	118.58	−67.13	−13.19	−79.90
**MU11**		−170.53	94.61	−16.95	−0.32	−59.61
**MU10**		−121.33	58.71	102.78	35.65	−19.85
**MU09**		−151.73	82.05	0.78	5.14	−50.07
**MU08**		−161.79	94.71	−17.08	8.33	−54.37
**MU07**		−147.18	83.90	9.33	10.88	−45.30
**MU06**		−156.48	86.23	10.88	5.69	−49.50
**MU05**		−149.89	87.21	−4.54	11.11	−48.11
**MU04**		−112.58	67.59	−3.10	20.37	−33.92
**MU03**		−105.32	57.70	32.04	16.73	−27.39
**MU02**		−100.02	58.76	6.01	22.12	−28.37
**MU01**		−70.19	62.81	−55.02	56.77	−19.25

**Table 9:** Model parameter list.

Item	Optimal Model	C_0_	C_0_+C	C/(C_0_+C)	Range (A)	R^2^
Annual average	exponential model	8800	41,850	0.790	2.325	0.903
Dry season	spherical model	119	439.9	0.729	0.632	0.629
Rainy season	exponential model	7700	52,280	3.426	0.853	0.944

**Table 10:** The relationships between soil and water quality in dry season.

		AP-Soil	SOM-Soil	TN-Soil	EC-Soil	pH-Soil
**pH-Water**	Pearson	−0.344	0.029	−0.071	0.352	0.431
	significance	0.149	0.907	0.772	0.14	0.065
**EC-Water**	Pearson	0.327	−0.534 *	−0.778 **	−0.643 **	−0.162
	significance	0.171	0.019	0	0.003	0.508
**DO-Water**	Pearson	0.429	0.034	0.275	−0.125	−0.364
	significance	0.067	0.891	0.255	0.611	0.125
**BOD-Water**	Pearson	−0.222	−0.144	−0.148	−0.172	0.044
	significance	0.361	0.555	0.546	0.482	0.859
**TP-Water**	Pearson	0.429	−0.093	−0.095	−0.412	−0.468 *
	significance	0.067	0.704	0.699	0.08	0.043
**NO_3_-N-Water**	Pearson	−0.606 **	0.418	0.469 *	0.756 **	0.446
	significance	0.006	0.075	0.043	0	0.056
**NO_2_-N-Water**	Pearson	−0.586 **	0.078	0.32	0.624 **	0.121
	significance	0.008	0.751	0.181	0.004	0.621
**NH_3_-N-Water**	Pearson	−0.552 *	0.296	0.454	0.605 **	0.259
	significance	0.014	0.219	0.051	0.006	0.284
**Pollution Composite Score**	Pearson	−0.357	0.483 *	0.738 **	0.638 **	0.137
	significance	0.134	0.036	0	0.003	0.576

** The correlation was significant at the 0.01 level (both sides). * The correlation was significant at the 0.05 level (both sides).

**Table 11:** Correlation of water quality and soil in rainy season.

		AP-Soil	SOM-Soil	TN-Soil	EC-Soil	pH-Soil
**pH-Water**	Pearson	−0.111	−0.331	−0.448	−0.425	−0.472 *
	significance	0.652	0.167	0.055	0.07	0.041
**EC-Water**	Pearson	−0.076	−0.207	−0.336	−0.446	−0.445
	significance	0.756	0.395	0.16	0.056	0.056
**DO-Water**	Pearson	−0.232	−0.029	0.068	0.001	−0.029
	significance	0.339	0.906	0.781	0.996	0.908
**BOD-Water**	Pearson	−0.216	−0.351	−0.251	−0.264	−0.18
	significance	0.375	0.14	0.3	0.275	0.46
**TP-Water**	Pearson	−0.311	−0.375	−0.534 *	−0.604 **	−0.562 *
	significance	0.195	0.113	0.018	0.006	0.012
**NO_3_-N-Water**	Pearson	0.529 *	0.117	0.121	0.209	0.235
	significance	0.02	0.633	0.622	0.39	0.333
**NO_2_-N-Water**	Pearson	0.274	0.542 *	0.659 **	0.644 **	0.600 **
	significance	0.256	0.017	0.002	0.003	0.007
**NH_3_-N-Water**	Pearson	0.041	0.346	0.544 *	0.767 **	0.677 **
	significance	0.869	0.147	0.016	0	0.001
**Pollution Composite Score**	Pearson	0.267	0.37	0.421	0.507 *	0.487 *
	significance	0.269	0.119	0.073	0.027	0.034

** The correlation was significant at the 0.01 level (both sides). * The correlation was significant at the 0.05 level (both sides).
